# Supplementary material for: A Novel Transgenic Reporter to Study Vertebrate Epigenetics
Source: bioRxiv. 2025 Apr 24:2025.04.21.649821. Preprint. [Version 1] doi: 10.1101/2025.04.21.649821 (PMC12190345; doi:10.1101/2025.04.21.649821)
Supplement: Supplement 1 [file media-1.pdf]

Supplementary Figures

Fig. S1

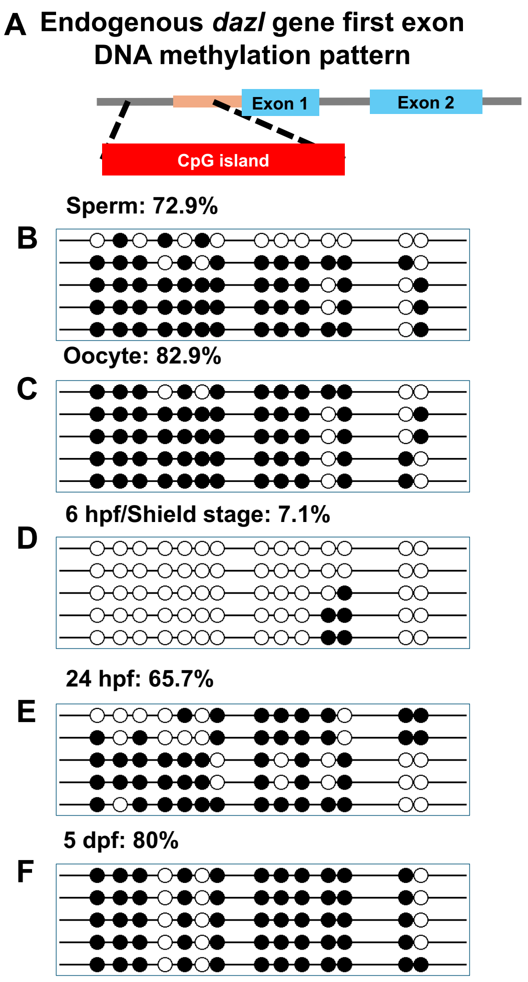

Fig. S1. Targeted bisulfite sequencing of the endogenous *dazl* CpG island.

(**A**) Schematic diagram showing position of the *dazl* CpG island upstream of the first exon tested for DNA methylation at different developmental stages and mature gametes. (**B-F**) bisulfite sequencing reads of *dazl* CpG island from the genomic DNA of mature sperm (**B**), mature oocytes (**C**), 6 hpf embryos (**D**), 24 hpf (**E**) and 5 dpf (**G**). Open circles represent unmethylated cytosine residues and filled circles represent methylated cytosine residues from CpG dinucleotides.

Fig. S2

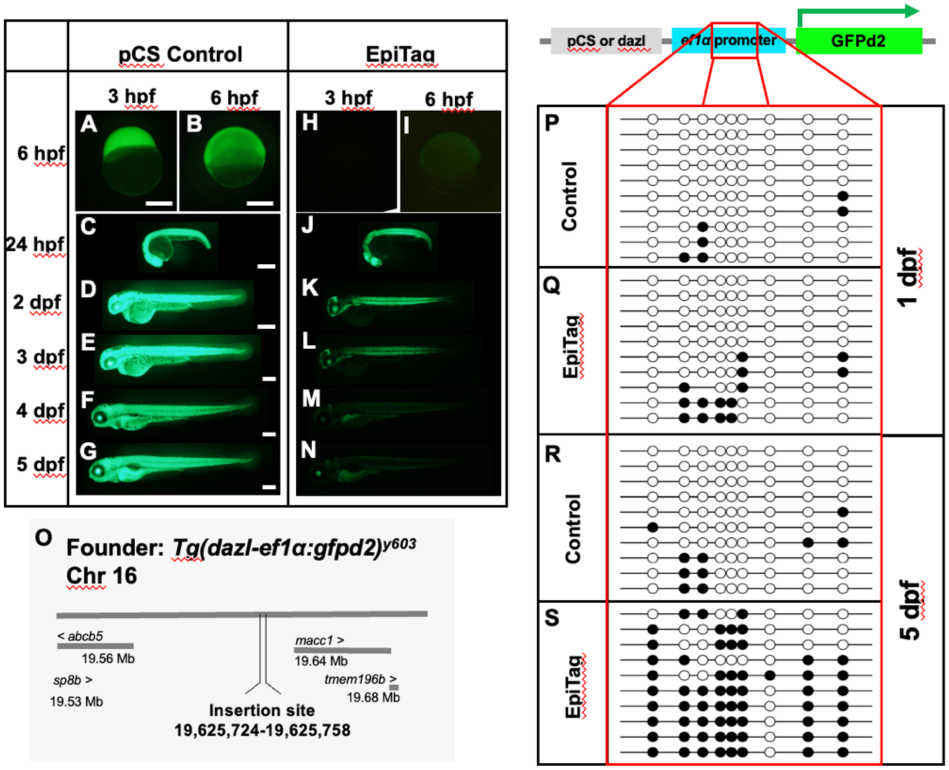

Fig. S2. Dynamic GFPd2 expression and methylation changes of the transgene insertion during embryonic and larval development.

(A-G) Green epifluorescence images of control-pCS, *Tg(pcs-ef1α:gfpd2)*^{y605} and **(H-N)** of EpiTag, *Tg(dazl-ef1α:gfpd2)*^{y603} embryonic and larval development. (**A,H**) 4 hpf, (**B,I**) 6 hpf, (**C,J**) 24 hpf, (**D,K**) 2 dpf, (**E,L**) 3 dpf, (**F,M**) 4 dpf and (**G,N**) 5 dpf. Scale bars = 200□m. (**O**) The EpiTag founder was isolated and mapped as described in the methods. Schematic diagram of the transgene and position of the *ef1α* promoter in the transgene. Red box noting approximate region of the *ef1α* promoter that was assayed for change in methylation. (**P-S**) Representative bisulfite sequencing reads of genomic DNA from 1 dpf embryos (**P,Q**) and 5 dpf larvae (**R,S**). Open circles represent unmethylated cytosine residues and filled circles represent methylated cytosine residues from CpG dinucleotides.

Fig. S3

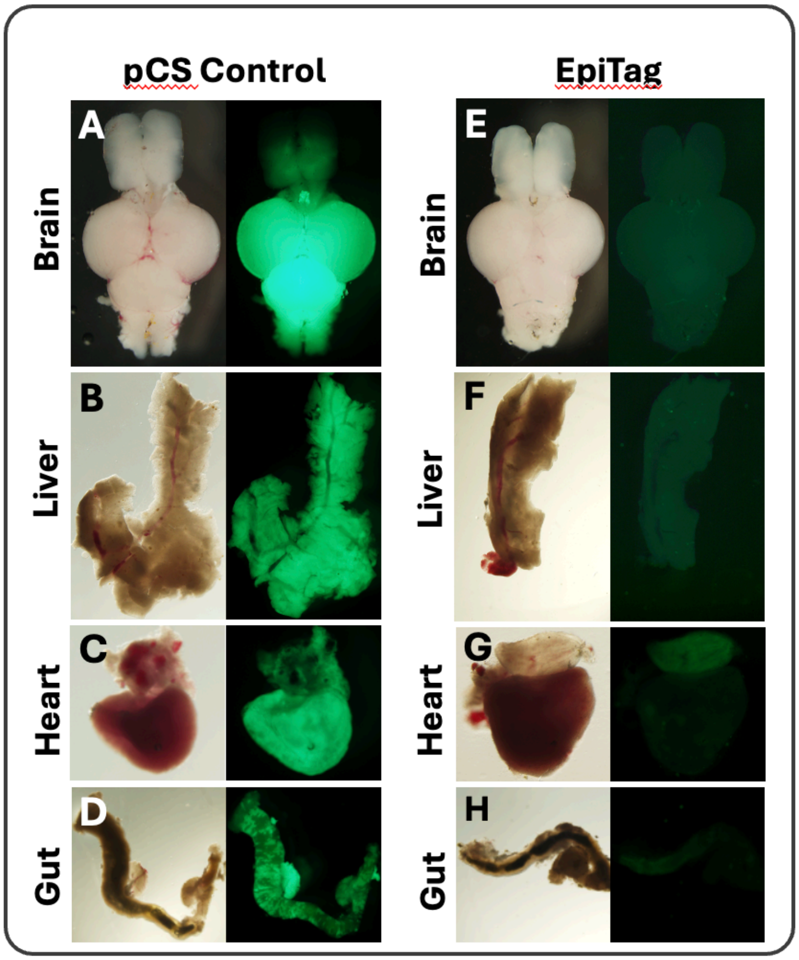

Fig. S3. EpiTag line silencing in internal adult organs.

(**A-H**) Paired bright field (left) and green epifluorescence (right) images of dissected internal organs from pCS Control (**A-D**) and EpiTag (**E-H**) transgenic animals. Brain (**A,E**), liver (**B,F**), heart (**C,G**) and gut (**D,H**) are shown. PCS control and EpiTag images were collected using the same settings for comparison.

Fig. S4

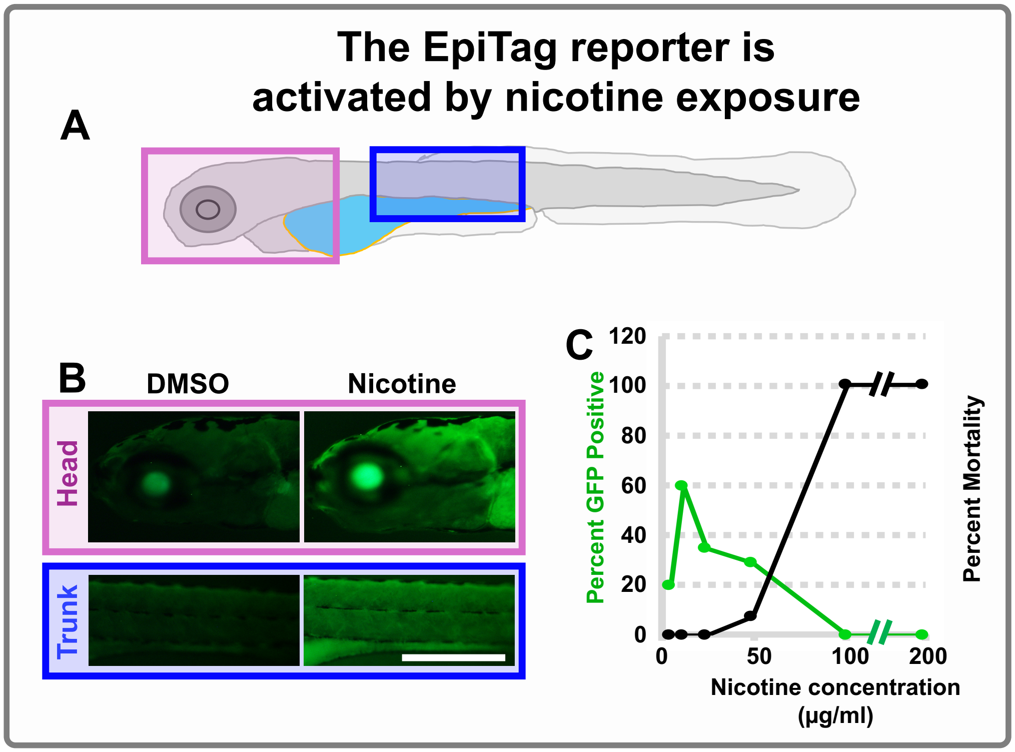

Fig. S4. Treatment of EpiTag larvae with nicotine results in GFPd2 activation at levels below those that induce mortality.

(A) Schematic diagram of a 5 dpf EpiTag larvae with magenta and blue boxes noting the approximate regions of the head and trunk imaged in panel B. (**B**) Green epifluorescence images of the heads (top) or trunks (bottom) of 5 dpf larvae treated with either DMSO (left) or nicotine (right). (**C**) Quantification of the percentage of GFP positive (green) or severely malformed/dead (black) 5 dpf EpiTag larvae after treatment from 3-5 dpf with the indicated concentrations of nicotine. Scale bar, 200 µm.

Fig. S5

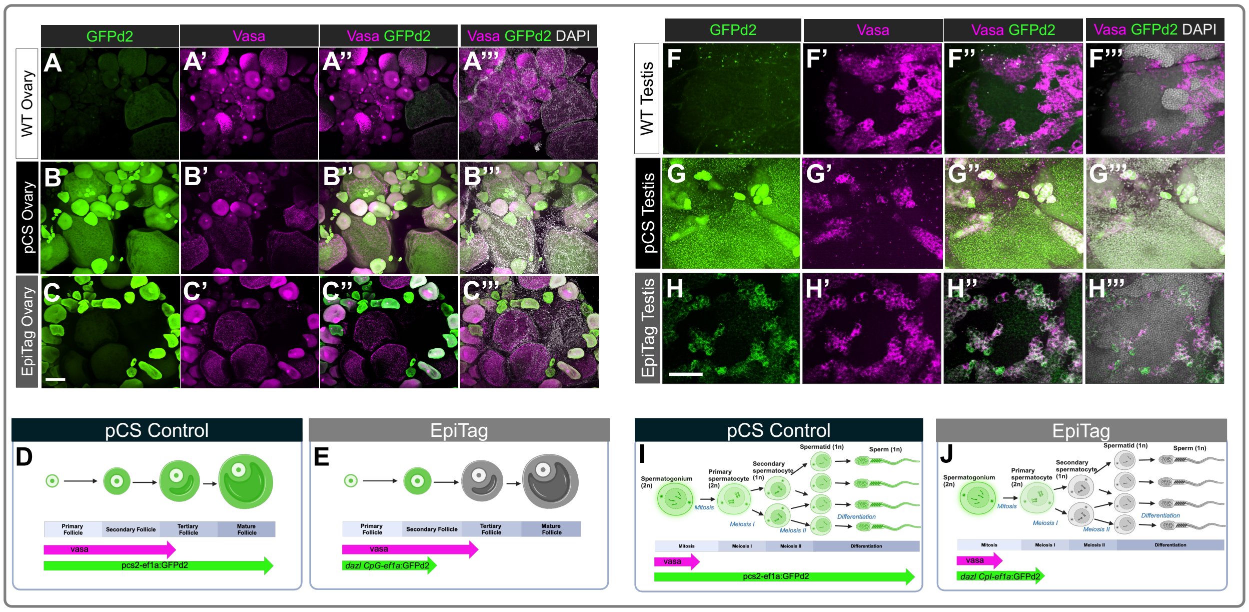

Fig. S5. GFPd2 expression in early stage oocytes and sperm colocalizes with Vasa

(**A-C**) Whole-mount ovary tissues of wild-type (**A**), *Tg(pcs-ef1α:gfpd2)*^{y605} Control (**B**), and *Tg(dazl-ef1α:gfpd2)*^{y603} EpiTag (**C**) adult females stained with *gfpd2* and *vasa* HCR probes and DAPI nuclear dye. Scale bar, 25 µm. (**D,E**) Schematic diagram showing different stages of oogenesis starting from the primary follicle and the corresponding GFPd2 expression patterns in pCS control (**D**) and EpiTag (**E**) fish. (**F-H**) Whole-mount testes tissues of wild-type (**F**), *Tg(pcs-ef1α:gfpd2)*^{y605} Control (**G**), and *Tg(dazl-ef1α:gfpd2)*^{y603} EpiTag (**H**) adult males stained with *gfpd2* and *vasa* HCR probes and DAPI nuclear dye. Scale bar, 25 µm (**I,J**) Schematic diagram showing different stages of spermatogenesis starting from the spermatogonium stage and the corresponding GFPd2 expression patterns in pCS Control (**I**) and EpiTag (**J**) fish.

Fig. S6

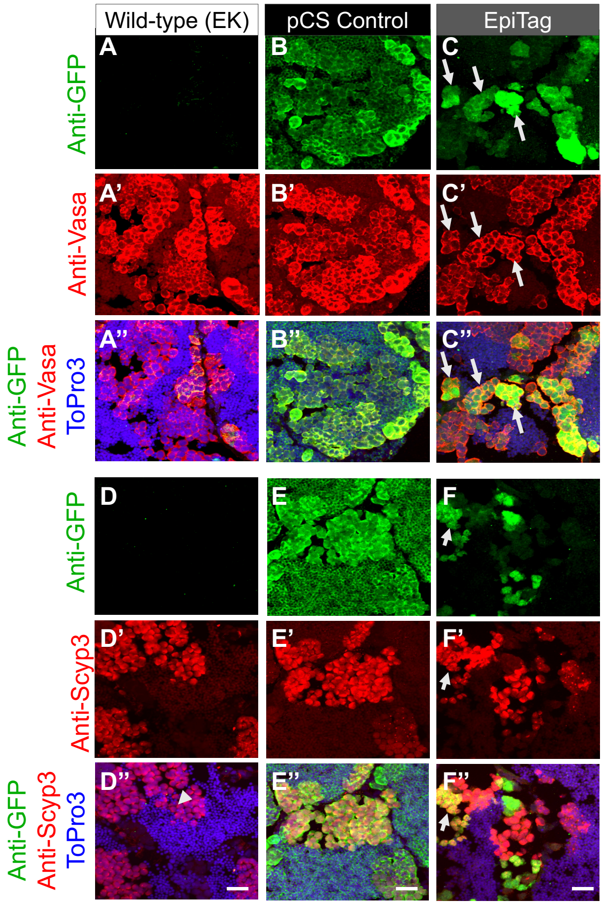

Fig. S6. GFPd2 reactivation during spermatogenesis

(**A-C''**) Immunohistochemical staining of cryosections from zebrafish testes probed for Anti-GFP (green) and anti-Vasa (red) and co-stained with ToPro3 nuclear dye (blue). (**D-F''**) Immunohistochemistry of cryosections from zebrafish testes probed for Anti-GFP (green) and anti-Scyp3 (red) and co-stained with ToPro3 nuclear dye (blue). Images shown are sections from wild-type **(A-A'', D-D'')**, pCS control (**B-B'', E-E"**) and EpiTag (**C-C", F-F"**) zebrafish testes. Scale bar, 20 µm.

Fig. S7

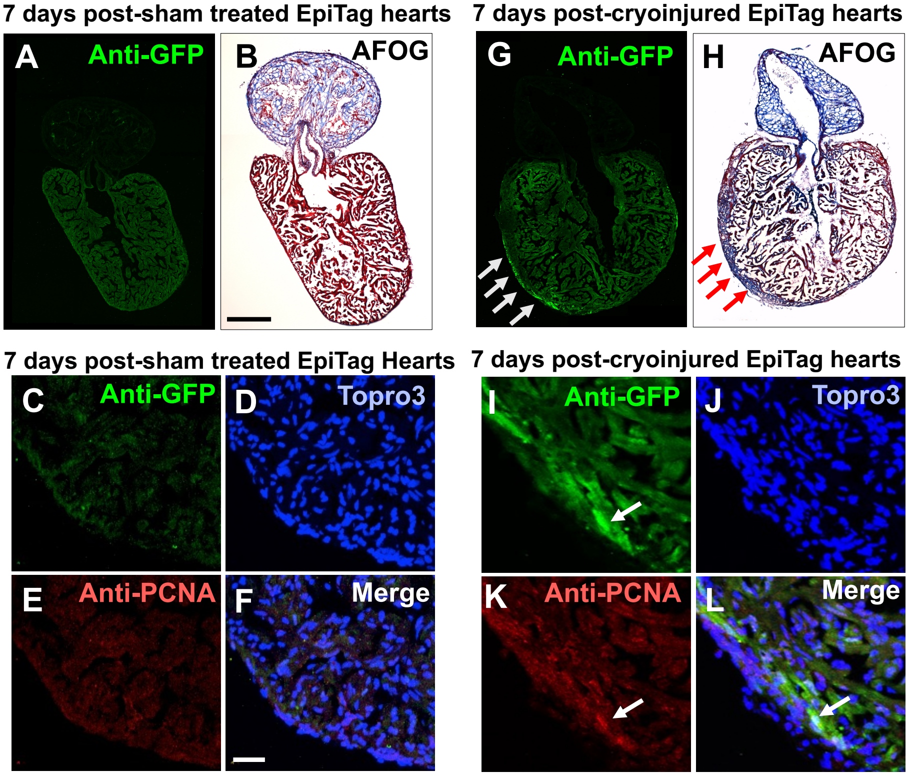

Fig. S7. GFPd2 reactivation during heart regeneration.

Immunohistochemical staining of cryosections of EpiTag zebrafish hearts either seven days post-sham treatment (**A-F**) or seven days post-cryoinjury (**G-L**). (**A,B**) and (**G,H**) are serial sections and panels (**C-F**) and panels (**I-L**) are showing the same sections. Anti-GFP (green) is shown in panels (**A,C,G,I**), anti-PCNA (red) is shown in panels (**E,K**), nuclei stained with ToPro3 (blue) are shown in panels (**D,J**) and merged images are shown in panels (**F,L**). AFOG staining is shown in panels (**B,H**). Scale bars = 200 µm (**A,B,G,H**) and 20 µm (**C-F,I-L**).
